# Woman’s Medical College

**Published:** 1883-04

**Authors:** 


### ARTICLE X.

### WOMAN'S MEDICAL COLLEGE.

The thirteenth annual commencement exercises of the Chicago Woman's Medical college were held February 13, at Hershey hall, in the presence of an overflowing house of friends of the young doctors. The exercises began promptly at 8 o'clock with an organ solo by Prof. Falk, succeeded by a short prayer by Rev. Samuel Fallows. Prof. William H. Byford, president of the college, then, in a few fitting words, presented diplomas and certificates to the following eighteen ladies, graduates of the class of 1883:

Mary Augusta Brown, Sarah Keyport Cummings, A.M., Elizabeth H. Cassell, Fannie Dickinson, Frank A. Groat, Elizabeth Sanders Holton, Laura North Hyde, Rebecca Alice Hartwell, Sophronia McCloud Lane, Virginia Mahoney, Julia Catherine Peffer, Georgia Sackett Ruggles, Charity Ann Saunders, Farima J. Shipp, Alice Summerfield, Avice Elida Smith, Mary A. White, Isabella Smith White, B. S.

The class valedictory address was read by Miss Virginia Mahoney. It was a clear, forcible effort, with but a moderate dash of the sentiment which generally adorns such farewell occasions. Miss Mahony gave a short review of the progress of medicine since the time of Æsculapius to the latter half of the nineteenth century. For the near future she predicted far greater and more brilliant advancement. Attention was called to the fact that the Chicago Woman's Medical college was the first of its kind in the West. The farewell to faculty and to the class was brief but well expressed.

Some of the class will go east, some north, and some as far west as China.

The regular faculty address was pronounced by Prof. Wadsworth as follows:

To provide for bodily comfort, to insure the soul's future happiness, and to attain knowledge are among the greater incentives to worldly industry. Now whatever may be the eccentricities of any one individual in these several directions the general development of the human mind is quite symmetrical. No one goes far ahead of the common understanding and maintains himself systematically. Science is classified knowledge. We can go back to pre-scientific as to pre-historic times when the general attainment of knowledge did not admit of classification in groups as at present, and if we observe the practice of medicine during these periods we shall see that there were few reasons or consistencies; but we have advanced and attained a considerable degree of knowledge in many directions. These remarks are made merely as a prelude to the subject around which our thoughts are to be gathered for this occasion, namely, the Principles and Practice of Medicine, minus or plus Physiology. and I define physiology as the science of essential organic movements with reference to functions. Æsculapius, the reputed son of Apollo, practiced more in surgery than in medicine, with very little knowledge of either and none whatever of physiology. Hippocrates hypothicated as "humors," blood, phlegm, yellow bile and black bile. He located blood in the heart, phlegm in the head, yellow bile in the liver, and black bile in the spleen and theorized that a surplus or disturbance of these "humors" caused all bodily ills; consequently bleeding and purging were his principal means of cure. Galen was an advocate of Hippocratic views. He believed that inflammation was the result of blood entering new areas of flesh; that when this was uncomplicated, it produced œdematoid disease; when accompanied by phlegm it produced phlegmonoid; by yellow bile erysipelatoid; by black bile schirrous disease, etc.

Upon these vague hypotheses he subjected his patients to the most rigorous confinement in heated and unventilated rooms to *sweat out* the offending humors. We need not discard him. He was wise for his time but he had not the slightest knowledge of physological processes. In the sixteenth century Paracelsus, a person of some acquirement of chemical knowledge, and who first introduced chemical remedies into medicines, declared that the human body was composed of mercury, sulphur and salt, and that in these substances all bodily disease had its origin. Mercury, as a volatile substance, gave rise to frenzy, madness, delerium etc.; sulphur produced fever, phlegmons, jaundice, etc.; while salts generated colic, stone gravel, gout and sciatica. His principal remedy was antimony, the physiological action of which he never even surmised.

The seventeenth century seems to be the earliest period in which observers contributed substantial material for a basis for physiological medicine. Harvey discovered the circulation of the blood, Malpighi extended his observations microscopically, and Bartholin, Steno, Wharton, Sylvius, Willis, Spigelius, Glisson, Peyer, De Graaf, Schneider and others did valuable service to science. During this period, however, medical practice was carried on upon the wildest hypotheses, and for the most part enshrouded in overpowering superstition. Nature had no province whatever. Sick bodies were cured by means as extrinsic and mysterious as those which saved souls, and the incentive to independent inqury by the few was held in check by the unreasoning ignorance of the many.

Pertinent to this subject the introduction at this time of inductive philosophy by Bacon aided greatly in preparing the general mind for the investigations and conclusions necessary to rational medicine. Early in the eighteenth century Stahl propounded the doctrine that the rational soul of man governs the whole bodily economy. That what we term the *vis medicatrix naturæ* was in reality the soul making resistance to bodily disturbances. Upon this hypothesis was based strenuous opposition to "strong medicine," and vigorous measures in treatment were decried by all adherents to the new doctrine. Antimony, opium, Peruvian bark, bleeding, purging, emetics etc., were discarded generally and expectation and timidity became characteristic. Immediately following the introduction of these views homœopathy with its *similia similibus curantur* and minimum dose list was introduced. The classic 10 and 10 divided into unthinkable attenuations making less disturbance in the body enhanced its favor with the soul and therethrough wrought wonderful cures minus physiological considerations.

During this century Haller discovered the property of the irritability in muscles and nerves, which gave a nucleus for new speculation and disjointed theories. Applied pathologically, disease was accounted to consist of spasm or debility. Every body sick was either sthenic or asthenic and therefore must be bled or stimulated. From this time on discoveries on physiological anatomy were frequent and important, but not until the beginning of the nineteenth century was there any one to conceive the idea of association in the theory and treatment of disease. Every theory based upon isolated facts or observation was run to its extreme, and the utmost confusion and contradiction of statement obtained. And who wonders? At present we have attained a much greater degree of physiological knowledge. Neither the investigations nor their achievements need be enumerated here.

Every medicine necessary to use has its physiological and therapeutical effect outlined, and none should be administered without an understanding of what is to be expected therefrom. And yet it must be said physiological considerations do not enter into the general practice of medicine as a ruling factor. The science, in its relation to principle and practice, is neither sufficiently taught nor comprehended, and the common people have not the slightest conception of such an idea. It is hardly an exaggeration to say that a thousand barrels of stuff in the form of patent medicine are swallowed annually by the people of Chicago at their own option, or on the advice of some friend. It was, in ancient times, the custom to expose the sick in public places and allow passers by to prescribe for them. The custom is modified but not much abated, and physicians are pestered by ignorant interference with their best remedial plans. As a matter of course legal medicine is not much in advance of common appreciation. In this State a person can legally practice medicine who has practiced ten years without medical education. One of these legalized doctors after attending a case of *dyssentery* ( as he pronounced it ) for two days, without a cure, prescribed peppermint water as his ultimatum of physiological medicine. Now should an intelligent M. D. enter protest against this doctor's practice he would be adjudged as jealous of his peer ( legally ), and the ignoramus would find plenty to support him.

The progress of medical science must, to a certain extent, be subservient to the common intelligence; but its advancement, nevertheless, must be achieved by persistent systematic research. Many suppose that medical sects, by rivalry, promote the interests of medicine; that homœopathy has modified regular medicine, abated bleeding, purging and heroic doses, and that eclecticism has abated the use of calomel. They have not, any more than the fly upon the wheel hub has turned the wheel, or the moonlight musings of lovesick boys and girls have advanced the science of astronomy. Sects are the incidents of an imperfect conception or the perversion of fundamental principles. Great advancements are continually being made independent of them and in spite of them, and they are left behind with their own littleness

The principles and practice of medicine *plus* physiology should be comprehended generally by practitioners, for the unphysiological practice of medicine is too nearly related to quackery, and in these days there should be no excuse for differences in this respect. Member of this class, remember thy physiology in the days of thy youth that thy patients may live long in the land.

The music of the evening was furnished by the Chicago female quartette. The floral presents made to the eighteen graduating young ladies were very numerous and beautiful.

